# Novel polymorphic and copy number diversity in the antibody IGH locus of South African individuals

**DOI:** 10.1007/s00251-024-01363-7

**Published:** 2024-12-04

**Authors:** Alaine A. Marsden, Martin Corcoran, Gunilla Karlsson Hedestam, Nigel Garrett, Salim S. Abdool Karim, Penny L. Moore, Dale Kitchin, Lynn Morris, Cathrine Scheepers

**Affiliations:** 1 SA MRC Antibody Immunity Research Unit (AIRU), University of the Witwatersrand, Johannesburg, South Africa; 2 Centre for HIV and STIs, HIV Virology Section, National Institute for Communicable Diseases (NICD), a Division of the National Health Laboratory Service (NHLS), Johannesburg, South Africa; 3 Department of Microbiology, Tumor and Cell Biology, Karolinska Institutet, Stockholm, Sweden; 4 Centre for the AIDS Programme of Research in South Africa (CAPRISA), University of KwaZulu-Natal, Durban, South Africa; 5 Discipline of Public Health Medicine, School of Nursing and Public Health, University of KwaZulu-Natal, Durban, South Africa; 6 Department of Epidemiology, Mailman School of Public Health, Columbia University, Columbia, NY USA

**Keywords:** Immunogenetics, Antibodies, Human genetics, Immunology

## Abstract

The heavy chain of an antibody is crucial for mediating antigen binding. IGHV genes, which partially encode the heavy chain of antibodies, exhibit vast genetic diversity largely through polymorphism and copy number variation (CNV). These genetic variations impact population-level expression levels. In this study, we analyzed expressed antibody transcriptomes and matched germline IGHV genes from donors from KwaZulu-Natal, South Africa. Amplicon NGS targeting germline IGHV sequences was performed on genomic DNA from 70 participants, eight of whom had matched datasets of expressed antibody transcriptomes. Germline IGHV sequencing identified 161 unique IGHV alleles, of which 32 were novel. A further 21 novel IGHV alleles were detected in the expressed transcriptomes of these donors. We also examined the datasets for CNV, uncovering gene duplications of 10 IGHV genes from germline sequencing and 33 genes in the expressed transcriptomes. Many of the IGHV gene duplications have not been described in other populations. This study expands our understanding of genetic differences in distinct populations and suggests the potential impact of genetic diversity on immune responses.

**Supplementary information:**

The online version contains supplementary material available at 10.1007/s00251-024-01363-7 .

## Introduction

Antibodies, or immunoglobulins, are a fundamental component of the adaptive immune response. The genes that encode the heavy chain of an antibody are all found within the immunoglobulin heavy chain (IGH) locus on chromosome 14 (Lefranc and Lefranc 2001). These include the variable (IGHV), diversity (IGHD), and joining (IGHJ) genes that are joined together through VDJ recombination to create the antigen-binding site. The IGH locus is one of the most polymorphic and structurally diverse loci in the human genome (Watson and Breden 2012). Single nucleotide variants (SNVs) across the locus create allelic diversity for IGH genes and large structural variants produce copy number variation (CNV) through the insertion, deletion, and duplication of multiple genes at a time (Watson et al. 2013). Amongst IGH genes, IGHV genes are the most numerous in the IGH locus, comprising up to 50 functional genes, 6 open reading frames, and 81 pseudogenes, divided into eight phylogenetic subgroups (IGHV1-8) (Lefranc and Lefranc 2001). For the purposes of this study, we focus on functional IGHV genes (IGHV1-7), avoiding pseudogenes and open reading frames.

Previous studies have demonstrated that polymorphic diversity and structural variation exhibit population-specific patterns (Watson et al. 2013; Avnir et al. 2016; Rodriguez et al. 2020). For example, the duplication of IGHV1-69 is more prevalent in African populations than in East Asian populations (Avnir et al. 2016). This is important because there is evidence that germline IGHV variation affects the expressed antibody transcriptomes, with CNV or polymorphism impacting IGHV gene usage (Avnir et al. 2016; Kenter et al. 2021; Pennell et al. 2023; Mikocziova et al. 2021a). Alterations in the expressed transcriptome frequently occur when an IGHV gene is involved in a duplication or complex event that alters copy number (such as for IGHV1-69, IGHV3-64D, IGHV5-10–1, IGHV1-8, and IGHV3-9, IGHV3-23) (Avnir et al. 2016; Rodriguez et al. 2023). Furthermore, SNVs in intergenic regions have been associated with alterations in gene expression across several genes, although the mechanism for this is unclear. For example, an A/G SNV occurring within the IGH locus (rs8008062), occurring 120 Kbp from any IGHV gene or known structural variant, has been shown to affect the expression of seven germline IGHV genes (Rodriguez et al. 2023). Of the seven genes, four (IGHV4-31, IGHV3-53, IGHV4-61, and IGHV1-69) were expressed at higher levels with a G nucleotide at the position as opposed to an A. Conversely, the remaining three genes (IGHV4-61, IGHV3-64, and IGHV3-66) were expressed at lower levels when a G nucleotide was found at the position in place of an A (Rodriguez et al. 2023). This study suggests that the SNV occurs in a regulatory region that modulates the chromatin formation of the locus, affecting the accessibility of IGHV genes to various enzyme complexes.

IGHV gene usage biases have also been described in a variety of infection and vaccination responses, resulting in convergent antibody responses across individuals. Influenza vaccine and infection responses frequently use the IGHV3-7, IGHV1-69, and IGHV4-39 genes. In SARS-CoV-2, there is evidence that IGHV3 family genes are commonly used in antibody responses (Nielsen et al. 2020; Mor et al. 2021). For HIV-1, broadly neutralizing antibody lineages often display the same IGHV gene usage across different epitopes, such as IGHV1-69 (MPER), IGHV4-34 (V3-Glycan), IGHV1-2 (CD4bs and V3-Glycan), and IGHV3-15 (MPER and V2-glycan) (Moyo et al. 2020; Doria-Rose et al. 2014; Richardson and Moore 2019; Zhou et al. 2015; Soto et al. 2016; Walker et al. 2009). If an IGHV gene is frequently observed in the transcriptomes of multiple individuals that have mounted a response against a pathogen or immunogen, it follows that the gene likely produces precursor B-cells with high affinity. Thus, having a gene duplication or genetic mutation that leads to overexpression of that particular IGHV gene may be beneficial in mounting a response.

In addition to gene usage biases, previous studies have shown that particular IGHV alleles can lead to more potent and/or broad responses to pathogens (Mikocziova et al. 2021a; Safonova et al. 2022). This has been demonstrated in influenza where there is a strong bias in the usage of IGHV1-69 and also key IGHV1-69 alleles that have a phenylalanine at position 54 produce more potent neutralizing responses (Avnir et al. 2016; Wheatley et al. 1950; Pappas et al. 2014; Jackson et al. 2014; Avnir et al. 2014). In HIV, a recent study has shown that VRC01-like CD4bs antibodies are produced more frequently in participants that express IGHV1-2*02 (Vaccine genetics of IGHV1-2 VRC01-class broadly neutralizing antibody precursor naïve human B cells - PubMed [Internet]. 2024).

We have previously sequenced the V-regions of functional IGHV genes in 28 participants from the CAPRISA cohorts (Scheepers et al. 2015). Here, we expand the scope of sequencing IGHV genes to include the regulatory parts of the gene, including the leader sequence and the recombination signal sequence (RSS). We developed an amplicon-based NGS approach to sequence the full IGHV gene from germline DNA, to identify novel IGHV alleles and genotypes for CNV. Furthermore, we mined existing transcriptomic data, sequenced from naive IgM B-cells of matched donors, to explore genetic diversity and its link to gene expression. Haplotype reconstruction was utilized from the transcriptomic data to examine CNV. Overall, we used a combination of both germline sequencing and transcriptome sequencing, within matched donors, to evaluate the impact of germline variation on the transcriptome.

## Methods and materials

### Study participants

This study utilized samples from the CAPRISA 002 and 004 cohorts. These cohorts consisted of black African women recruited in the KwaZulu-Natal province of South Africa. CAPRISA 002 examined acute HIV infection (Loggerenberg et al. 2008), while CAPRISA 004 was a clinical trial of tenofovir gel in preventing HIV (Abdool Karim et al. 2010). There is overlap between the participants that were involved in both studies, thus representing a combined cohort. A total of 70 women from both CAPRISA cohorts participated in this study, of whom 58 were participants living with HIV, including the 28 donors in the previous study by Scheepers et al. (2015), and 12 were participants without HIV. Informed consent was obtained from all participants for sample collection and storage for future studies.

### Amplification of germline IGHV genes

Genomic DNA was extracted from stored peripheral blood mononuclear cells (PBMC) using the Wizard® Genomic DNA Purification Kit (Promega; Madison, WI, USA). Primers were designed to amplify the full IGHV gene, including the regulatory regions, for the first six IGHV subgroups (Supplementary Table 1). The IGHV7 subgroup of genes failed to yield full-length amplicons and was omitted from this study. However, we have previously reported on IGHV7 in this population, which was done utilizing a different set of primers designed to target within the V-region of the IGHV gene (Scheepers et al. 2015). The primers used in this study differ in that they bind outside the entire IGHV gene, in the untranslated regions because our aim was to produce full-length sequences. The forward primer for each subgroup bound before leader part 1 of the respective IGHV gene and the reverse primer bound after the RSS. Amplicon sizes ranged from ~ 570 bp for IGHV1 genes to ~ 880 bp for IGHV4 genes. Each PCR reaction contained 15.75 µL of molecular grade water, 5 µL of 5 × Platinum SuperFi Buffer, 0.5 µL of a dNTP mix (10 µM each), 1.25 µL of forward primer (10 µM), 1.25 µL of reverse primer (10 µM), and 0.25 µL of Platinum SuperFi Polymerase (Thermo Fisher Scientific; Waltham, MA, USA) (2.5 U/µL). To this mix, 1 µL of DNA (10 ng/µL) was added. The cycling conditions had an initial denaturation of 30 s at 98 °C. There were 35 cycles of denaturation at 98 °C for 30 s, annealing for 10 s, and extension at 72 °C for 1 min. The final extension was for 2 min at 72 °C. Annealing temperatures varied by primer pair (see Supplementary Table 1). Each sample was run in duplicate to ensure adequate coverage and minimize primer biases.

### NGS library preparation and sequencing of germline IGHV genes

The IGHV amplicons were purified using AMPure XP beads (Beckman Coulter; Brea, CA, USA). Beads were added to the amplicons at a 1:0.75 ratio, to ensure the removal of small DNA fragments (< 200 bp). IGHV1 amplicons were sequenced on the Illumina MiSeq platform due to MiSeq being more appropriate for the amplicon size (570 bp) and the greater sequencing depth that can be produced in a run. This was necessary due to the large number (*n* = 21) of genes in this subgroup and the high allelic diversity of IGHV1 genes, such as IGHV1-69. The other subgroups were sequenced using the PacBio Sequel platform (IGHV2 through IGHV6); these were then pooled using a gene-appropriate ratio (Supplementary Table 1), where the final concentration for each subgroup was proportional to the number of genes in each reaction. All amplicons were indexed with participant-specific molecular barcodes. IGHV1 amplicons, sequenced on the Illumina MiSeq, were indexed with the Nextera Flex indices (Illumina; San Diego, CA, USA) using the Failsafe PCR kit (Epicentre; Madison, WI, USA) in a limited 11 cycle PCR. Each indexing reaction contained 10 µL of Nextera index mix, 25 µL of Failsafe PCR 2✕ PreMix, 13.5 µL of water, 0.5 µL Failsafe PCR enzyme mix, and 1 µL of amplicon DNA. IGHV2 to IGHV6 were indexed using Pacific Biosciences (Menlo, CA, USA) barcodes and the Platinum SuperFi kit, using the same protocol as initial amplification except with the indexes as primers.

Sequencing library quality assessment and sequencing were performed by the NICD Sequencing Core. Briefly, the amplicon library concentration was measured on a Qubit® 3.0 Fluorometer (Invitrogen; Waltham, MA, USA) and the size of the amplicons were then visualized using the 4200 TapeStation (Agilent Technologies; Santa Clara, CA, USA). The purified amplicon libraries were normalized to 2 nM and then sequenced on the Illumina MiSeq platform using the Illumina MiSeq v3 kit with a 10% PhiX spike in, to obtain 2 × 300 bp paired-end sequences. The library preparation for PacBio libraries was done using the SMRTbell Template Prep Kit (v.1.0) (Pacific Biosciences). Sequencing was carried out on PacBio® Sequel system using SMRT cell 1 M with a movie time of 600 min, pre-extension time of 90 min using Sequel Binding Kit (v.2.1), Sequel Sequencing Plate (v.2.1) and loading was done by diffusion. The resulting subreads were demultiplexed and the circular consensus sequences (CCSs) were generated using the “ccs” command (0.9999 minimum predicted accuracy and 3 minimum passes) on SMRT Link (v.6.0) (Pacific Biosciences).

### Sequence analysis of germline IGHV sequences

Paired-end MiSeq FASTQ files were paired using PEAR (v0.9.8) (Zhang et al. 2014). PacBio CCS FASTQ files did not require this step. Primers were then removed from all reads, from both platforms, using CutAdapt (v3.5) (Martin 2011). The FASTQ files were converted to FASTA using the FASTX toolkit (Lab 2010). Removal of low-quality reads (Q score < 33), dereplication, and removal of low-abundance reads were done using VSEARCH (v2.22.1) (Rognes et al. 2016). Processed sequences were assigned gene and allele annotations using a custom Julia script developed for this analysis utilizing modules and packages from BioJulia (https://github.com/NICD-AIRU/AMarsden_IGHV_Analysis_2024). Briefly, the V-region of the IGHV genes were compared to a custom database, created using reference sequence data from IMGT (imgt.org, accessed in Jan 2023) (Lefranc et al. 2015; Manso et al. 2022; Lefranc 2014) and IgPdb (https://cgi.cse.unsw.edu.au/~ihmmune/IgPdb), using BLAST (v2.12). The BLAST output was then parsed to select the closest match with the highest sequence identity for the sequence and assign a gene and allele for the sequence. Pairwise alignment was then performed to identify SNVs (single nucleotide variants) in the regulatory regions of the gene using reference leader, intron, and RSS sequences from IMGT, noting the region and frequency of SNVs across the gene. The script also examined the number of unique alleles per person for each gene; if there were more than two unique alleles assigned to a gene, that gene was assumed to be duplicated in that participant.

### Analysis of antibody transcriptome datasets

Of the 70 CAPRISA donors used for the germline IGHV sequencing, eight had donor-matched bulk IgM transcriptomic sequencing, obtained pre-infection, that were utilized in this study. Amongst the eight, two have been published in a study examining public clonotypes in longitudinal samples from HIV-infected individuals (Setliff et al. 2018). The sequence data from the previously published study was produced with the methodology outlined in the original paper (Setliff et al. 2018). Briefly, unbiased amplification of VDJs was performed using a multiplex approach, with forward primers designed to amplify all heavy chain regions by targeting the framework-1 region. These underwent quality assessment before sequencing on an Illumina MiSeq. The unpublished data in this study was produced using a 5′ multiplex method to amplify full VDJs, using a different primer set which targets the leader sequence (Vázquez Bernat et al. 2019).

All transcriptomic sequencing data were processed from paired-end Illumina MiSeq reads that were paired using PEAR. Sequence data was identified and annotated using PyIR, a python wrapper for IgBlast (v1.20.0) (Soto et al. 2020). Novel allele inference was performed using IgDiscover (v1.01) (Corcoran et al. 2016). Individual haplotypes were reconstructed with RabHit, based on the allele assignment and inference of novel alleles, initially made by IgDiscover using IGHJ6 as a gene anchor, which was heterozygous in 4/8 participants and yielded reliable results (results with confidence scores above 3 according to RabHit (v0.2.5) (Peres et al. 2019). Other IGHJ genes, IGHJ1-IGHJ5, were assessed, but fewer participants were heterozygous with sufficient expression for RabHit for these genes (IGHJ1:0, IGHJ2:0, IGHJ3:0, IGHJ4:2, IGHJ5:0). Custom Julia scripts (https://github.com/NICD-AIRU/AMarsden_IGHV_Analysis_2024) were used to compare the outputs of the transcriptomic and germline sequencing analyses. In brief, the scripts count the number of unique alleles per haplotype. The expressed allele calls were compared with the germline allele calls for the participants to establish concordance. The novel IgDiscover calls are compared to existing novel alleles from the germline sequencing and IgPDb alleles. We assessed the impact of duplication on expression by comparing unique VDJ counts between haplotypes with duplication to haplotypes without duplication, on a per gene basis. This was conducted on a gene-wise basis to avoid the effect of variable expression between genes. Duplications were also excluded from this analysis if they had a read count under 50 or if the Bayesian significance factor (k-value) was under 3.6, deeming the inference as unsubstantial (see Supplementary Table 7).

## Results

### Novel alleles identified by germline and bulk IgM transcriptome sequencing

**Fig. 1:**
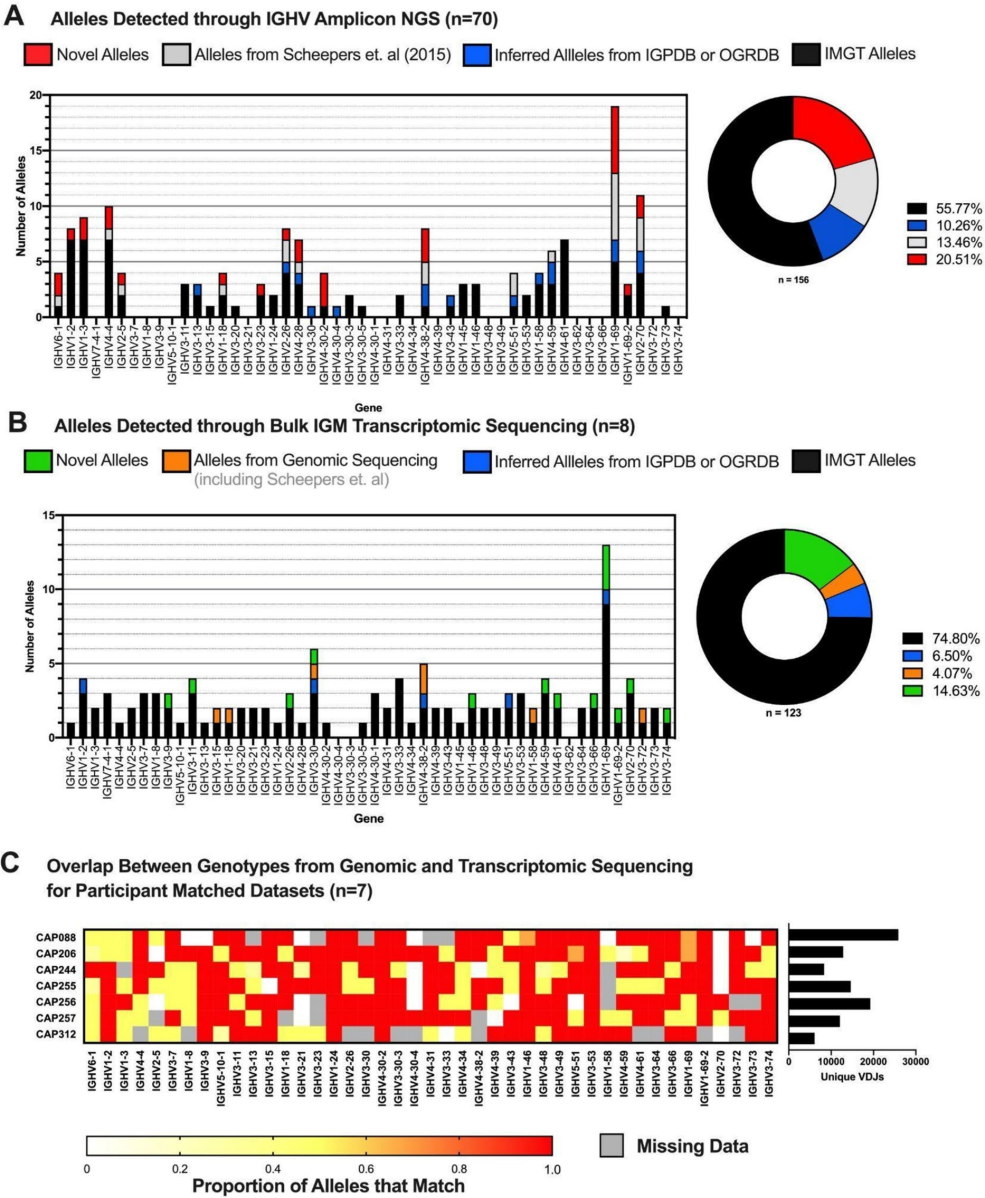
Distribution of IGHV alleles identified through transcriptomic and germline sequencing. **A** Alleles identified from germline NGS, coloured by the alleles that were novel (red), previously identified in Scheepers et al. (2015) (grey), matched alleles in the IgPDb/OGRDB (blue), and IMGT (black) databases. **B** Alleles identified from transcriptome sequencing of bulk IgM, coloured by the categorization of the alleles with novel alleles (green), the alleles which match previous genomic sequencing from Scheepers et al. (2015) (orange), inferred alleles from other databases (blue), and IMGT alleles (black). **C** Heatmap showing the overlap between the genomic and transcriptomic sequencing datasets for seven matched participants. Participant 228 was excluded due to low VDJ count. Grey represents where either approach is lacking data for that gene. The sequencing depth from the transcriptome sequencing is represented by the bar graph to the right that shows the number of unique VDJs identified per participant

Full-length germline sequencing of IGHV genes from 70 individuals revealed 161 alleles (Fig. 1A, Supplementary Table 2) across 33 IGHV genes. While 56% (*n* = 87) of these alleles matched known sequences in the IMGT database (indicated in black), 21% (*n* = 32) were novel (indicated in red). Novel sequences and their identity to known IMGT alleles are detailed in Supplementary Table 3. The majority of the remaining alleles (13%, *n* = 21) matched those previously identified from the same donors, indicated in grey (Scheepers et al. 2015). The final 10% (*n* = 16) of alleles matched inferred sequences from the IgPDb or OGRDB databases (indicated in blue). Unique alleles were determined using full-length sequences, inclusive of all regions of the IGHV gene. This created instances where V-regions between alleles were identical but there were differences in other regions such as the intron or leader sequence (Supplementary Table 4).

Some genes had less sequences than other genes, as evidenced by lower or non-existent allele calls, particularly those genes within the IGHV3 family. Due to the fact that we were able to amplify them in the transcriptomic data from the same donors (Fig. 1B) and have amplified them in previous germline sequencing (using different primers) (Scheepers et al. 2015), it is unlikely that the missing sequences result from germline deletions. Therefore, the missing sequences likely result from PCR biases due to primer multiplexing, with the primers favouring some genes over others. It is also possible that unknown nucleotide variation lays in the primer binding sites.

We next aimed to examine the expressed antibody transcriptome for further novel variants and determine if the novel alleles described in this study can be observed in expressed antibodies. From the larger cohort of 70 individuals, eight had matching bulk IgM-expressed datasets comprising more than 5 million total sequences. We performed germline inference to identify IGHV alleles in these expressed datasets (Fig. 1B, Supplementary Table 2). There were a greater number of IGHV genes observed using this approach, with alleles being identified for 48/50 IGHV genes (compared to 33 in the germline data). In total, 123 alleles were identified; the majority (75%, *n* = 92) of which matched known alleles in the IMGT database (black, Fig. 1B). Most non-IMGT alleles were novel (green), making up 15% (*n* = 18) of alleles; identity to known IMGT alleles is shown in Supplementary Table 3. A small proportion (7%, *n* = 8, blue) of the sequences matched inferred alleles from other studies found in IgPDb or OGRDB (Lees et al. 2020), while a minority matched alleles from genomic sequencing in either this or the previously study on these donors (4%, *n* = 5, orange).

We compared the allele assignments for the donors that had high VDJ counts and found strong concordance between the germline and transcriptomic sets (Fig. 1C). This analysis focussed on genes where there was sufficient overlap between the datasets, excluding genes where there was substantial missing data. One of the participants (CAP228) was also excluded due to low VDJ counts. In genes where coverage from both techniques for a participant was high, concordance was strong (> 80%). The discrepancy between the datasets, where concordance was low, is partly due to large differences in IGHV coverage between the two methods. Furthermore, discordance may arise in calls between the datasets (the white boxes) as the result of the different way alleles are identified. In the transcriptome, not all IGHV alleles, or even certain IGHV genes, may be expressed, hampering the ability to efficiently discriminate true alleles from somatically mutated sequences. For example, IGHV2-70 is highly discordant in our dataset. This likely results from the low levels at which IGHV2-70 is expressed which makes it difficult to correctly infer alleles for this gene. For CAP312, there are two genomic novel alleles (IGHV6-1*03_C288T and IGHV6-1*01_C288T) which differ to the transcriptome (which shows IGHV6-1*01). It is possible that the genomic alleles contain mutations that prevent their expression or were not expressed at high levels at the particular time point sampled. Furthermore, the mutation lies towards the end of the transcript sequence where uncertainty from inference exists due to variable v sequence lengths. Thus, the discrepancy likely arises from technical limitations in the inference process. The primers utilized in the germline approach may sit in regions where unknown polymorphic diversity lays, affecting their efficacy, as opposed to the primers in the transcriptomic approaches which target conserved regions within the IGHV gene.

### Leader sequence variation in full-length germline IGHV sequences

**Fig. 2:**
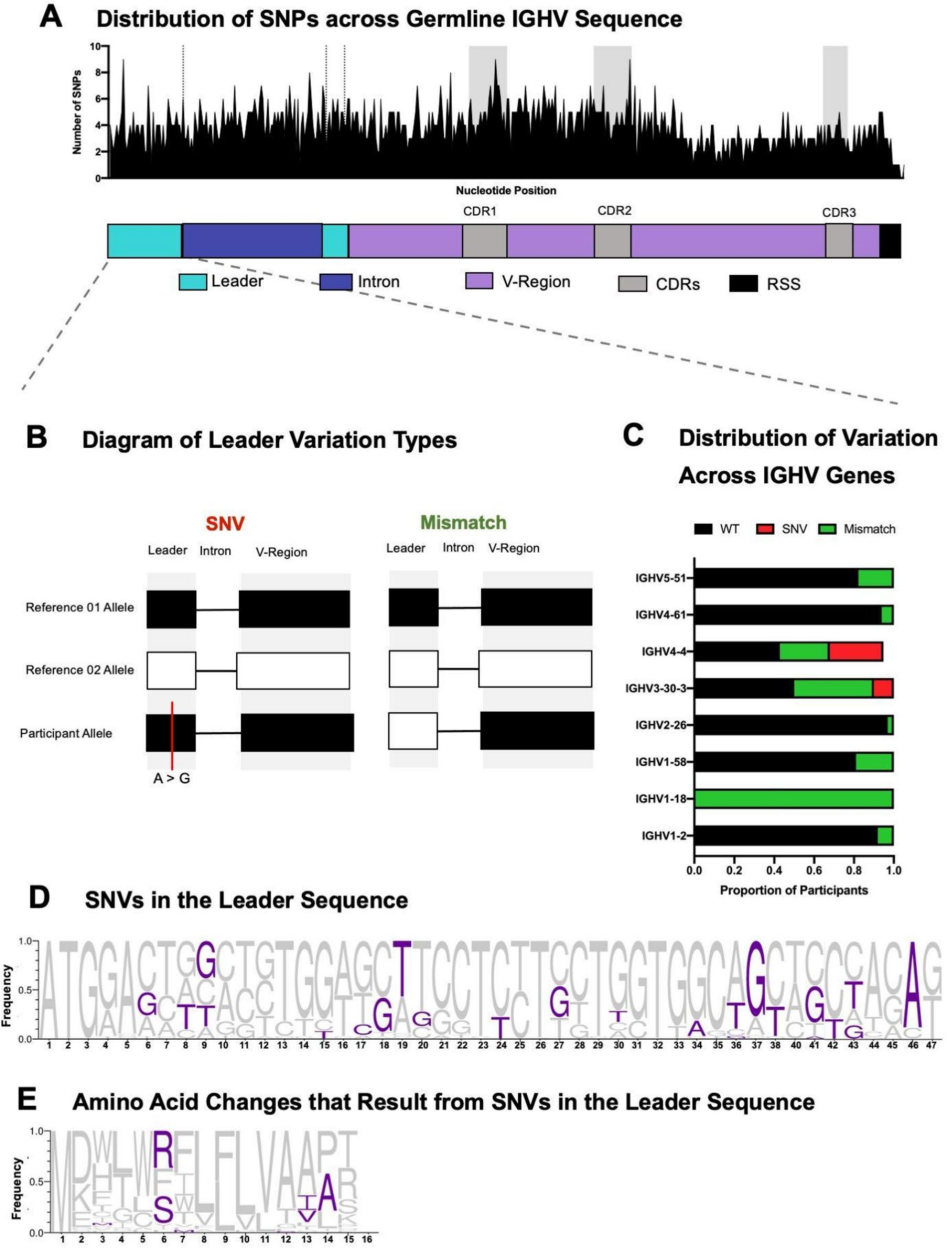
Genetic diversity in the leader sequence of IGHV genes from germline sequencing. **A** SNPs across the full-length IGHV gene. The peaks represent the number of single nucleotide variations across the IGHV gene. The various regions of the gene are highlighted both on the graph and below in a schematic. It should be noted that CDR3 in this figure does not represent the whole CDR3 in a final antibody sequence as this is the germline IGHV portion of the CDR3, distinct from the CDR3 in the protein of an antibody that arises from the junction of the V, D, and J genes during somatic recombination. **B** Schematic representation of differing forms of leader part 1 sequence diversity; alignment is demonstrated in Supplementary Fig. 1. **C** Distribution of leader sequence diversity across specific IGHV genes with SNV in red and Mismatch in green. **D** Logogram representing the relative frequency of novel leader sequence variations when contrasted against reference leader sequence data (from IMGT). The novel bases are represented in purple. **E** Logogram of translated leader sequence data, representing novel amino acid changes (purple) as the result of leader sequence diversity

Using full-length germline sequences, we examined the distribution of nucleotide diversity across the whole gene, including the leader sequence and V-region (Fig. 2A). This revealed variation across all parts of the IGHV gene, not just in the V-region, which is the most extensively studied.

Within the first part of the leader region (before the intron, Supplementary Table 5), we identified two types of variation: one involving single nucleotide variation (SNVs) and the other in which the leader sequence is represented in IMGT but is associated with a different V-region than what has been detected in these sequences, resulting in a mismatch between the expected leader and V-regions. This latter variation resulted in a different assignment of the leader sequence from the V-region identity, which we termed mismatch (Fig. 2B). The mismatch may arise from nucleotide variation, such as SNVs, or these variants are the product of homologous recombination. The different variations are also represented in alignment form in Supplementary Fig. 1. These analyses excluded novel alleles and alleles that matched truncated data from IgPDb, as there is no reference leader sequence for these alleles for comparison to identify SNVs or mismatch. The mismatch variation was observed in 20% of alleles and present in eight genes, in contrast to the SNV which was detected in 6% of alleles and only present in two genes. Notably, all IGHV1-18 alleles had mismatched leaders.

Despite the variations being across different genes and alleles, there was commonality in the variable nucleotide positions (Fig. 2D). For several positions (nucleotides: A19T, A37G, and C46A) across the leader, novel mutations were more prevalent than the nucleotides from reference IMGT leader sequences. Several of these leader variants lead to amino acid changes (Fig. 2E), which may have implications for antibody expression that warrant further investigation.

### Gene duplication confirmed by reconstructed haplotypes

**Fig. 3:**
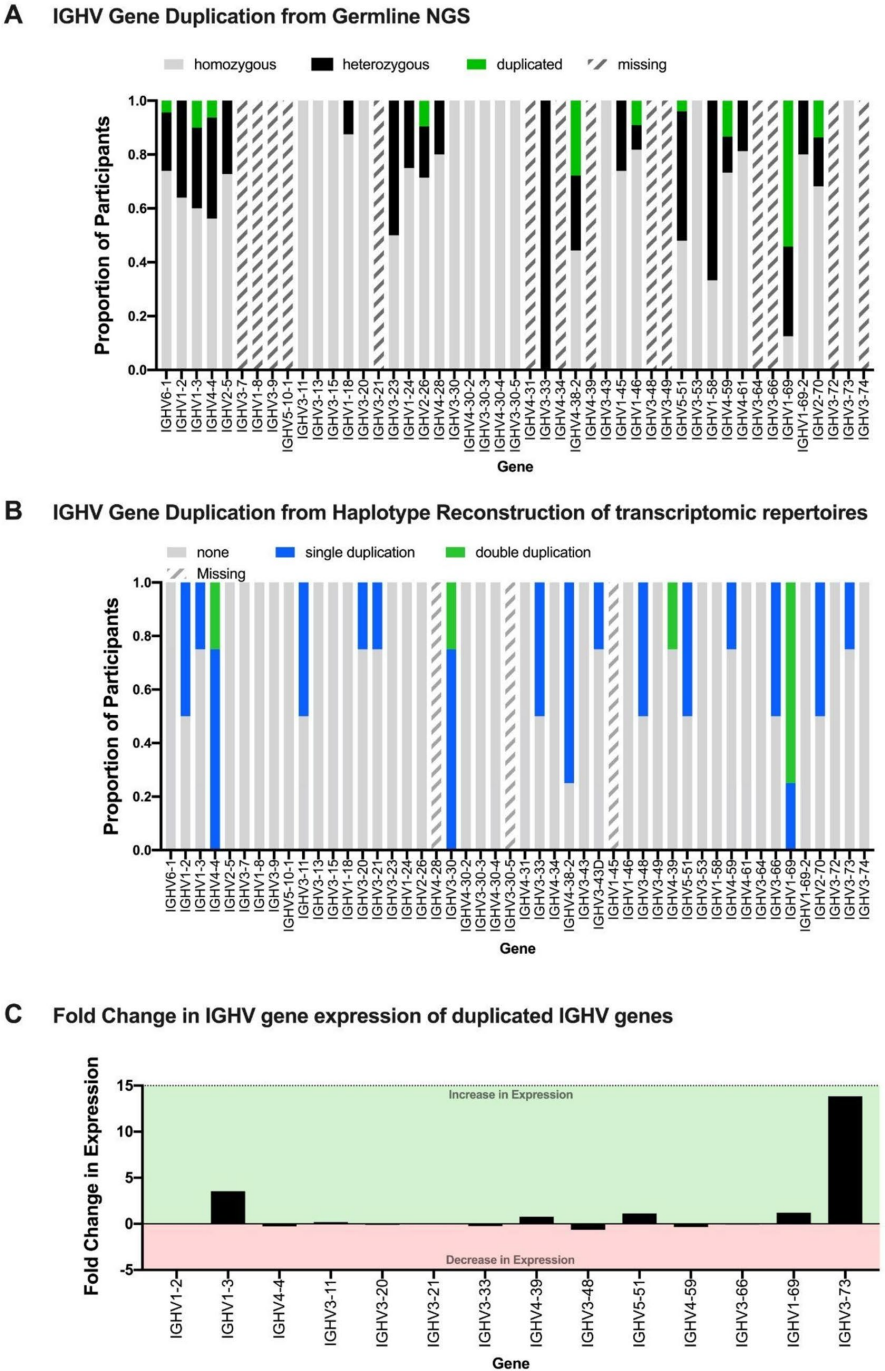
Gene duplication in IGHV genes. **A** Duplication of IGHV genes detected in germline sequencing of 70 participants. The bars represent the proportion of the participants who were homozygous (grey), heterozygous (black), or were duplicated (green) for a given gene. **B** CNV detected from haplotype reconstruction of donor-matched expressed IgM data. The bars represent the proportion of the participants who contained no duplication (grey), a single haplotype duplication (blue), and duplications on both haplotypes that were resolved (green). **C** Fold change in expression between haplotypes with duplication and those without duplication. Expression was calculated by taking the difference between the relative proportions a particular gene was expressed as in association with either haplotype

CNV analysis was performed on both germline and transcriptomic datasets to examine the presence of gene duplication. For the germline NGS data (Fig. 3A), duplication was called when there were more than two unique alleles for a gene in a participant. From this, we estimated duplication in 10/33 IGHV genes. Gene deletion, conversely, was difficult to estimate with accuracy as a single unique allele may indicate homozygosity rather than a deletion. Furthermore, no alleles detected for a particular gene may be the result of poor sequencing coverage; thus, deletions were excluded from this analysis. A caveat of this approach is that two unique alleles may exist on one chromosome while the other chromosome contains a deletion. Furthermore, this approach cannot take into account that duplications may be the result of completely identical alleles; thus, we could be underestimating duplications in this dataset. The participants that contained duplications and the alleles that contributed to duplication are noted in Supplementary Table 6. It should be noted that the analysis was performed on a per gene basis, examining participants that had coverage of that gene. Thus, the proportions do not represent a proportion of the total population but those that had sequences for a particular gene. For example, there were few participants that had IGHV3-33 sequences which resulted in two alleles identified (Fig. 1A); however, both of these participants were heterozygous for IGHV3-33.

Gene duplication has been reported in nine IGHV genes in other studies (Watson et al. 2013; Avnir et al. 2016). Of those genes, four (IGHV1-46, IGHV4-59, IGHV1-69, and IGHV2-70) were also duplicated in individuals in our population. The most commonly duplicated gene was IGHV1-69, which was duplicated in more than half of the population, while the other genes were less commonly duplicated (20%, *n* = 14). Furthermore, six IGHV genes (IGHV6-1, IGHV1-3, IGHV4-4, IGHV2-26, IGHV4-38–2, and IGHV5-51) demonstrate novel duplication, not reported in other CNV studies. The most commonly duplicated gene amongst the novel duplications was IGHV4-38–2 (27%, *n* = 19), showing prevalence similar to genes with reported duplications. The remaining genes had less than 10% prevalence in the population, although this level of prevalence is still striking.

We performed haplotype reconstruction on the transcriptome data to analyse the presence of CNV. Briefly, haplotype reconstruction leverages the process of VDJ recombination to examine which alleles of a particular IGHV gene are expressed with a particular IGHJ allele (in this study, as with many, IGHJ6, served as the anchor). As genes that undergo VDJ recombination exist on the chromosome, a haplotype can be reconstructed from examining which alleles exist together in certain VDJ combinations. We were able to resolve CNV on a chromosome level by examining the number of alleles assigned for each gene to a particular haplotype. As a result of higher IGHV gene coverage in the transcriptomic data, compared to the germline sequencing, we identified duplications in 18 genes (Fig. 3B). The duplications often consisted of the same IGHV gene alleles on both chromosomes. Furthermore, through haplotype analysis, we were able to resolve whether duplications occurred on a single chromosome or both chromosomes. Duplications on both chromosomes were present in 4/50 IGHV genes in this dataset. These indicate that there is inheritance of these duplications from both parents, suggesting a wider prevalence of IGHV gene duplication in our population. Due to the complexity innate to assigning alleles to particular genes, there may be overrepresentation of duplication for certain genes. An example of this is IGHV3-30 where allele *18 is identical to an allele of IGHV3-30–5. Indeed this allele is implicated for several participants as part of IGHV3-30 duplication and thus may not represent true duplication. Furthermore, no alleles were assigned to IGHV1-69D and IGHV2-70D which are duplicated genes and highly homologous to IGHV1-69 and IGHV2-70. Therefore, it is possible that some alleles assigned to the non- “D” genes are in fact alleles of those genes. However, this would not necessarily change the detection of duplication within this dataset. This also holds true for the genomic data produced in this study. This analysis is further complicated by the differences in expression between alleles of IGHJ6, as in the case of CAP088 and CAP256. The haplotyping process of RabHit was adjusted to account for this but may however be a limitation. Nevertheless, these data provide information on potential CNV events that can be validated by genomic techniques.

Unique VDJ counts were normalized as a proportion of the transcripts to control for variable VDJ counts between participants. These were then used to calculate a fold change in expression between haplotypes that did not have a duplication and those that did. With the reconstructed haplotypes from transcriptome data, we were able to connect the presence of a duplication with differences in expression and validate duplication observed previously in germline sequencing (Fig. 3C). While most of the genes demonstrate marginal fold changes as the result of gene duplication (> onefold change), there were six genes that demonstrate a much more distinct increase in gene expression when duplicated (IGHV1-3: 5.2 fold, IGHV3-9: 3.4 fold, IGHV5-51: 1.4 fold, IGHV1-69: 1.2 fold, IGHV2-70: 3.1 fold, IGHV3-73: 10.5 fold). This is consistent with what has been reported in other studies, where duplicated genes such as IGHV1-69 demonstrate biased usage (Avnir et al. 2016; Kenter et al. 2021; Pennell et al. 2023). The broad range (1.4–10.5 fold change) in expression levels amongst these genes suggests that duplication alone is not the only causative factor in enhanced expression indicating a more complex basis for this phenomenon. Some of these differences in expression could be explained by biased allele usage, which has been reported in several genes (Ohlin 2021). This may particularly be the case for the duplicated haplotypes for IGHV1-3 which harbour an 01 allele which is expressed much more highly than the 02 alleles present in the haplotypes that lack duplication. While this is the only allele with reported usage biases that is present in the haplotypes of the dataset, it remains possible that as yet unknown allelic biases may be contributing to the observed differences in expression.

## Discussion

Here we examined the genetic variation in both the IGHV sequences of a South African population, using multiple approaches. We observed significant levels of novel genetic diversity including in the leader peptide sequence, a functionally important portion of the gene that modulates expression. Importantly, many of these novel alleles were expressed as part of the antibody transcriptome, suggesting that they are utilized in the adaptive immune response. Furthermore, gene duplications were observed in the majority of the sequenced genes with some evidence of changes in expression levels as a result of duplication.

There are currently more than 300 alleles recorded in IMGT and other databases (Lefranc et al. 2015; Manso et al. 2022; Lefranc 2014; Lees et al. 2020). Through sequencing of 70 African donors, we have contributed an additional 32 novel IGHV alleles. We have also provided full-length sequences for 37 previously truncated alleles, allowing more confident inclusion of those sequences within human germline reference data. High levels of novel variation are consistent with several recent studies that examine the IGH locus (Watson and Breden 2012; Watson et al. 2013; Rodriguez et al. 2020; Corcoran et al. 2016; Gidoni et al. 2019; Hardt et al. 2023; Boyd et al. 1950). Other studies have examined African participants; however, our population yielded different alleles to those identified in largely Yoruban samples (Watson et al. 2013; Scheepers et al. 2015). It is probable that IG loci follow the population sub-structures identified more broadly in African genomes (Choudhury et al. 2020; Sengupta et al. 2020; Tau et al. 2017; May et al. 2013). Notably, however, the samples used in this study come from a single geographic population (KwaZulu-Natal, South Africa) and likely do not represent the broader genetic diversity across South Africa or the Southern African region. Further studies in cohorts across Southern Africa, and Africa more broadly, are likely to reveal novel genetic diversity.

Transcriptomic sequencing data derived from a subset of 8 of these individuals revealed further novel genetic diversity and explored linkage to gene expression. These results included confirmation that novel alleles discovered are expressed and contribute to a functional antibody response. Furthermore, the transcriptome sequencing showed better coverage across IGHV genes than the germline sequencing. This is likely the result of the sequencing method being more unbiased than the germline approach. The transcriptomic approach targeted highly conserved regions (framework 1 and the leader sequence) within the gene, whereas the germline sequencing used primers that target intergenic regions outside of the gene. Unknown polymorphisms in these intergenic regions may have impacted the ability of these primers to amplify all IGHV genes reliably. Our approach could be improved by a more complete understanding of the genetic diversity in intergenic regions in the IGH locus for Southern African individuals. Newer approaches, like hybrid capture described in Rodriguez et al. (2023), can provide complete characterization of both structural and allelic diversity in the IGH locus and could be applied in future studies.

There has been a large increase in the number of antibody transcriptomic studies being performed globally. Many of the data for these studies are housed in public databases such as iReceptor (Corrie et al. 2018) and VDJbase (Omer et al. 2020) and there are rigorous processes for the validation of novel alleles described from inference (Ohlin et al. 2019). This study indicates the utility of leveraging previously generated data to examine genetic diversity in IGH genes and creates a useful link to expression in the antibody transcripts. Meta-studies enable a deeper understanding of human genetic variation in the IG loci.

Full IGHV sequences were produced by amplicon sequencing, allowing for the examination of the leader sequence upstream of the variable gene. Similar to variable genes, substantial diversity was uncovered in the leader sequences. This has also been reported in the heavy chain (IGH) and light chain (IGK, IGL) loci of other populations (Mikocziova et al. 2020; Zhu et al. 2021; Mikocziova et al. 2021b; Huang et al. 2021). This study confirms that this phenomenon occurs in many of the same genes discussed in previous studies (IGHV1-18, IGHV1-58, IGHV2-26, IGHV4-4, IGHV4-61, IGHV5-51), inclusive of the mismatch variation described here. Furthermore, many variants identified in these studies are present in our dataset. For example, many of the IGHV4-4 alleles identified in this study demonstrate variation at IMGT position −31 in the leader between G and C, which is consistent with what has been reported in previous studies (Mikocziova et al. 2020; Zhu et al. 2021; Mikocziova et al. 2021b; Huang et al. 2021). These studies broadly focussed on European populations and here we described similar variation in African populations, suggesting that this phenomenon is common across different human populations. The uncovered mismatches in allele assignment between the leader and the variable regions may be the result of nucleotide variation that simply matches other alleles or could possibly represent a recombination event between alleles due to high sequence homology. It is probable that while there may be specific demographic variants, leader sequence variation is more widely common in the human IG loci. Furthermore, there are considerations around allelic nomenclature that arise from this data, as IGHV gene alleles are assigned on identity around the V-region of the gene. If this leader sequence variation is common, we need to consider how matching V-regions with differing leader sequences may in fact be different alleles of that particular gene. This may especially be the case where leader sequence differences alter gene expression. The amino acid variants uncovered in this study may, with further exploration, help to explain how different genes and alleles are expressed at variable levels within a population.

IGHV gene duplication was commonly found in this population, consistent with what has been reported in other African populations (Watson et al. 2013; Avnir et al. 2016). While discrepancies exist in the CNV detected between the genomic and transcriptomic datasets, this can be attributed to the differences in gene coverage between the datasets and also the discrepancy in sample size (70 genomic participants versus 4 participants that were haplotyped with IGHJ6). Furthermore, differences between the observed CNV between the datasets could also result from different allele calls from the analysis pipelines utilized in this study. The presence of CNV on both chromosomes has also been demonstrated in other populations and studies (Rodriguez et al. 2020; Chaisson et al. 2019). Hybridization capture allows for more complete characterization of the germline IGHV repertoire. It provides an alternative approach to examining genetic diversity that overcomes some of the limitations of this study, which can be the result of biases in expression. This study serves as an indicator of potential CNV variants that may be validated using such genomic means. Some of these copy number variants lead to alterations in expression which have been reported previously resulting in enhanced antibody levels (Avnir et al. 2016).

Overall, in this study, we have demonstrated that inferred structural variants and allele calls can closely match germline variation, supporting the use of antibody transcriptomic sequencing as a proxy for germline study. Through the combination of techniques that target the germline and examine the transcriptome, we uncovered a vast array of genetic diversity which spans from polymorphic diversity and CNV. This study emphasizes the need for more immunogenetic studies with paired germline and transcriptomic datasets as a way to investigate complex genetic phenomena and their impact on the antibody response.

## Supplementary information

Below is the link to the electronic supplementary material.

Supplementary file1 (DOCX 18 KB)

Supplementary file2 (XLSX 12 KB)

Supplementary file3 (XLSX 8 KB)

Supplementary file4 (XLSX 14 KB)

Supplementary file5 (XLSX 8 KB)

Supplementary file6 (XLSX 6 KB)

Supplementary file7 (XLSX 9 KB)

Supplementary file8 (XLSX 57 KB)

## Data Availability

Raw reads from both germline sequencing and unpublished transcriptome sequencing are available on SRA under PRJNA983809. The previously published transcriptomic data is available on SRA under PRJNA415492. Processed IGHV sequences from germline sequencing and inferred from expressed repertoires have been deposited on GenBank under the following accession numbers: OR078942–OR079165.

## References

[CR1] Abdool Karim Q, Abdool Karim SS, Frohlich JA, Grobler AC, Baxter C, Mansoor LE et al (2010) Effectiveness and safety of tenofovir gel, an antiretroviral microbicide, for the prevention of HIV infection in women. Science 329(5996):1168–1174 20643915 10.1126/science.1193748 PMC3001187

[CR2] Avnir Y, Tallarico AS, Zhu Q, Bennett AS, Connelly G, Sheehan J et al (2014) Molecular signatures of hemagglutinin stem-directed heterosubtypic human neutralizing antibodies against influenza A viruses. PLoS Pathog 10(5):e1004103 24788925 10.1371/journal.ppat.1004103 PMC4006906

[CR3] Avnir Y, Watson CT, Glanville J, Peterson EC, Tallarico AS, Bennett AS et al (2016) IGHV1-69 polymorphism modulates anti-influenza antibody repertoires, correlates with IGHV utilization shifts and varies by ethnicity. Sci Rep 6(1):20842 26880249 10.1038/srep20842 PMC4754645

[CR4] Boyd SD, Gaëta BA, Jackson KJ, Fire AZ, Marshall EL, Merker JD et al (1950) 2010 Individual variation in the germline Ig gene repertoire inferred from variable region gene rearrangements. J Immunol Baltim Md 184(12):6986–92 10.4049/jimmunol.1000445 PMC4281569 20495067

[CR5] Chaisson MJP, Sanders AD, Zhao X, Malhotra A, Porubsky D, Rausch T et al (2019) Multi-platform discovery of haplotype-resolved structural variation in human genomes. Nat Commun 16(10):1784 10.1038/s41467-018-08148-z PMC6467913 30992455

[CR6] Choudhury A, Aron S, Botigué LR, Sengupta D, Botha G, Bensellak T et al (2020) High-depth African genomes inform human migration and health. Nature 586(7831):741–748 33116287 10.1038/s41586-020-2859-7 PMC7759466

[CR7] Corcoran MM, Phad GE, Bernat NV, Stahl-Hennig C, Sumida N, Persson MAA et al (2016) Production of individualized V gene databases reveals high levels of immunoglobulin genetic diversity. Nat Commun 7:13642 27995928 10.1038/ncomms13642 PMC5187446

[CR8] Corrie BD, Marthandan N, Zimonja B, Jaglale J, Zhou Y, Barr E et al (2018) iReceptor: a platform for querying and analyzing antibody/B-cell and T-cell receptor repertoire data across federated repositories. Immunol Rev 284(1):24–41 29944754 10.1111/imr.12666 PMC6344122

[CR9] Doria-Rose NA, Schramm CA, Gorman J, Moore PL, Bhiman JN, DeKosky BJ et al (2014) Developmental pathway for potent V1V2-directed HIV-neutralizing antibodies. Nature 509(7498):55–62 24590074 10.1038/nature13036 PMC4395007

[CR10] Gidoni M, Snir O, Peres A, Polak P, Lindeman I, Mikocziova I et al (2019) Mosaic deletion patterns of the human antibody heavy chain gene locus shown by Bayesian haplotyping. Nat Commun 10(1):628 30733445 10.1038/s41467-019-08489-3 PMC6367474

[CR11] Hardt U, Corcoran MM, Narang S, Malmström V, Padyukov L, Karlsson Hedestam GB (2023) Analysis of IGH allele content in a sample group of rheumatoid arthritis patients demonstrates unrevealed population heterogeneity. Front Immunol 14:1073414 36798124 10.3389/fimmu.2023.1073414 PMC9927645

[CR12] Huang Y, Thörnqvist L, Ohlin M (2021) Computational inference, validation, and Analysis of 5'UTR-leader sequences of alleles of immunoglobulin heavy chain variable genes. Front Immunol 12:730105. 10.3389/fimmu.2021.730105 10.3389/fimmu.2021.730105 PMC8521166 34671351

[CR13] Jackson KJL, Liu Y, Roskin KM, Glanville J, Hoh RA, Seo K et al (2014) Human responses to influenza vaccination show seroconversion signatures and convergent antibody rearrangements. Cell Host Microbe 16(1):105–114 24981332 10.1016/j.chom.2014.05.013 PMC4158033

[CR14] Kenter AL, Watson CT, Spille JH (2021) Igh locus polymorphism may dictate topological chromatin conformation and V gene usage in the Ig repertoire. Front Immunol 12:682589 34084176 10.3389/fimmu.2021.682589 PMC8167033

[CR15] Lab H (2010) FASTX toolkit. Hannon Lab Cold Spring Harbor, NY

[CR16] Lees W, Busse CE, Corcoran M, Ohlin M, Scheepers C, Matsen FA IV et al (2020) OGRDB: a reference database of inferred immune receptor genes. Nucleic Acids Res 48(D1):D964–D970 31566225 10.1093/nar/gkz822 PMC6943078

[CR17] Lefranc MP, Giudicelli V, Duroux P, Jabado-Michaloud J, Folch G, Aouinti S et al (2015) IMGT, the international ImMunoGeneTics information system 25 years on. Nucleic Acids Res 43:D413-422 25378316 10.1093/nar/gku1056 PMC4383898

[CR18] Lefranc MP (2014) Immunoglobulin and T cell receptor genes: IMGT(®) and the birth and rise of immunoinformatics. Front Immunol 5:22. 10.3389/fimmu.2014.00022 10.3389/fimmu.2014.00022 PMC3913909 24600447

[CR19] Lefranc M-P, Lefranc G (2001) The immunoglobulin factsBook, Academic Press, p 458

[CR20] Manso T, Folch G, Giudicelli V, Jabado-Michaloud J, Kushwaha A, Nguefack Ngoune V et al (2022) IMGT® databases, related tools and web resources through three main axes of research and development. Nucleic Acids Res 50(D1):D1262–D1272 34875068 10.1093/nar/gkab1136 PMC8728119

[CR21] Martin M (2011) Cutadapt removes adapter sequences from high-throughput sequencing reads. EMBnet J 17(1):10–2

[CR22] May A, Hazelhurst S, Li Y, Norris SA, Govind N, Tikly M et al (2013) Genetic diversity in black South Africans from Soweto. BMC Genomics 14:644 24059264 10.1186/1471-2164-14-644 PMC3850641

[CR23] Mikocziova I, Gidoni M, Lindeman I, Peres A, Snir O, Yaari G et al (2020) Polymorphisms in human immunoglobulin heavy chain variable genes and their upstream regions. Nucleic Acids Res 48(10):5499–5510 32365177 10.1093/nar/gkaa310 PMC7261178

[CR24] Mikocziova I, Greiff V, Sollid LM (2021a) Immunoglobulin germline gene variation and its impact on human disease. Genes Immun 22(4):205–217 34175903 10.1038/s41435-021-00145-5 PMC8234759

[CR25] Mikocziova I, Peres A, Gidoni M, Greiff V, Yaari G, Sollid LM (2021) Germline polymorphisms and alternative splicing of human immunoglobulin light chain genes. iScience 103192. 10.1016/j.isci.2021.103192 10.1016/j.isci.2021.103192 PMC8517844 34693229

[CR26] Mor M, Werbner M, Alter J, Safra M, Chomsky E, Lee JC et al (2021) Multi-clonal SARS-CoV-2 neutralization by antibodies isolated from severe COVID-19 convalescent donors. PLoS Pathog 17(2):e1009165 33571304 10.1371/journal.ppat.1009165 PMC7877634

[CR27] Moyo T, Kitchin D, Moore PL (2020) Targeting the N332-supersite of the HIV-1 envelope for vaccine design. Expert Opin Ther Targets 24(6):499–509 32340497 10.1080/14728222.2020.1752183 PMC7530370

[CR28] Nielsen SCA, Yang F, Jackson KJL, Hoh RA, Röltgen K, Jean GH et al (2020) Human B cell clonal expansion and convergent antibody responses to SARS-CoV-2. Cell Host Microbe 28(4):516-525.e5 32941787 10.1016/j.chom.2020.09.002 PMC7470783

[CR29] Ohlin M (2021) Poorly expressed alleles of several human immunoglobulin heavy chain variable genes are common in the human population. Front Immunol 11:603980. 10.3389/fimmu.2020.603980 10.3389/fimmu.2020.603980 PMC7943739 33717051

[CR30] Ohlin M, Scheepers C, Corcoran M, Lees WD, Busse CE, Bagnara D et al (2019) Inferred allelic variants of immunoglobulin receptor genes: a system for their evaluation, documentation, and naming. Front Immunol 10:435 30936866 10.3389/fimmu.2019.00435 PMC6431624

[CR31] Omer A, Shemesh O, Peres A, Polak P, Shepherd AJ, Watson CT et al (2020) VDJbase: an adaptive immune receptor genotype and haplotype database. Nucleic Acids Res 48(D1):D1051–D1056 31602484 10.1093/nar/gkz872 PMC6943044

[CR32] Pappas L, Foglierini M, Piccoli L, Kallewaard NL, Turrini F, Silacci C et al (2014) Rapid development of broadly influenza neutralizing antibodies through redundant mutations. Nature 516(7531):418–422 25296253 10.1038/nature13764

[CR33] Pennell M, Rodriguez OL, Watson CT, Greiff V (2023) The evolutionary and functional significance of germline immunoglobulin gene variation. Trends Immunol 44(1):7–21 36470826 10.1016/j.it.2022.11.001

[CR34] Peres A, Gidoni M, Polak P, Yaari G (2019) RAbHIT: R antibody haplotype inference tool. Bioinforma Oxf Engl 35(22):4840–4842 10.1093/bioinformatics/btz481 31173062

[CR35] Richardson SI, Moore PL (2019) The antibody response in HIV-1-infected donors. Curr Opin HIV AIDS 14(4):233–239 31033730 10.1097/COH.0000000000000559 PMC7324637

[CR36] Rodriguez OL, Gibson WS, Parks T, Emery M, Powell J, Strahl M et al (2020) A novel framework for characterizing genomic haplotype diversity in the human immunoglobulin heavy chain locus. Front Immunol 11:2136 33072076 10.3389/fimmu.2020.02136 PMC7539625

[CR37] Rodriguez OL, Safonova Y, Silver CA, Shields K, Gibson WS, Kos JT et al (2023) Genetic variation in the immunoglobulin heavy chain locus shapes the human antibody repertoire. Nat Commun 14(1):4419 37479682 10.1038/s41467-023-40070-x PMC10362067

[CR38] Rognes T, Flouri T, Nichols B, Quince C, Mahé F (2016) VSEARCH: a versatile open source tool for metagenomics. PeerJ 4:e2584 27781170 10.7717/peerj.2584 PMC5075697

[CR39] Safonova Y, Shin SB, Kramer L, Reecy J, Watson CT, Smith TPL et al (2022) Variations in antibody repertoires correlate with vaccine responses. Genome Res 32(4):791–804 35361626 10.1101/gr.276027.121 PMC8997358

[CR40] Scheepers C, Shrestha RK, Lambson BE, Jackson KJL, Wright IA, Naicker D et al (2015) Ability to develop broadly neutralizing HIV-1 antibodies is not restricted by the germline Ig gene repertoire. J Immunol 194(9). 10.4049/jimmunol.1500118 10.4049/jimmunol.1500118 PMC4513073 25825450

[CR41] Sengupta D, Choudhury A, Fortes-Lima C, Aron S, Whitelaw G, Bostoen K et al (2020) Genetic-substructure and complex demographic history of South African Bantu speakers. 10.1038/s41467-021-22207-y 10.1038/s41467-021-22207-y PMC8027885 33828095

[CR42] Setliff I, McDonnell WJ, Raju N, Bombardi RG, Murji AA, Scheepers C et al (2018) Multi-donor longitudinal antibody repertoire sequencing reveals the existence of public antibody clonotypes in HIV-1 infection. Cell Host Microbe 23(6):845-854.e6 29861170 10.1016/j.chom.2018.05.001 PMC6002606

[CR43] Soto C, Ofek G, Joyce MG, Zhang B, McKee K, Longo NS et al (2016) Developmental pathway of the MPER-directed HIV-1-neutralizing antibody 10E8. PLoS One 11(6):e0157409 27299673 10.1371/journal.pone.0157409 PMC4907498

[CR44] Soto C, Finn JA, Willis JR, Day SB, Sinkovits RS, Jones T et al (2020) PyIR: a scalable wrapper for processing billions of immunoglobulin and T cell receptor sequences using IgBLAST. BMC Bioinformatics 21(1):314 32677886 10.1186/s12859-020-03649-5 PMC7364545

[CR45] Tau T, Wally A, Fanie TP, Ngono GL, Mpoloka SW, Davison S et al (2017) Genetic variation and population structure of Botswana populations as identified with AmpFLSTR Identifiler short tandem repeat (STR) loci. Sci Rep 7(1):6768 28754995 10.1038/s41598-017-06365-y PMC5533702

[CR46] Vaccine genetics of IGHV1-2 VRC01-class broadly neutralizing antibody precursor naïve human B cells - PubMed [Internet] (2024) Available from: https://pubmed.ncbi.nlm.nih.gov/34489473/10.1038/s41541-021-00376-7 PMC8421370 34489473

[CR47] van Loggerenberg F, Mlisana K, Williamson C, Auld SC, Morris L, Gray CM et al (2008) Establishing a cohort at high risk of HIV infection in South Africa: challenges and experiences of the CAPRISA 002 acute infection study Ugarte-Gil CA, editor. PLoS One 3(4):e1954 18414658 10.1371/journal.pone.0001954 PMC2278382

[CR48] Vázquez Bernat N, Corcoran M, Hardt U, Kaduk M, Phad GE, Martin M et al (2019) High-quality library preparation for NGS-based immunoglobulin germline gene inference and repertoire expression analysis. Front Immunol 10:660 31024532 10.3389/fimmu.2019.00660 PMC6459949

[CR49] Walker LM, Phogat SK, Chan-Hui PY, Wagner D, Phung P, Goss JL et al (2009) Broad and potent neutralizing antibodies from an African donor reveal a new HIV-1 vaccine target. Science 326(5950):285–289 19729618 10.1126/science.1178746 PMC3335270

[CR50] Watson CT, Breden F (2012) The immunoglobulin heavy chain locus: genetic variation, missing data, and implications for human disease. Genes Immun 13(5):363–373 22551722 10.1038/gene.2012.12

[CR51] Watson CT, Steinberg KM, Huddleston J, Warren RL, Malig M, Schein J et al (2013) Complete haplotype sequence of the human immunoglobulin heavy-chain variable, diversity, and joining genes and characterization of allelic and copy-number variation. Am J Hum Genet 92(4):530–546 23541343 10.1016/j.ajhg.2013.03.004 PMC3617388

[CR52] Wheatley AK, Whittle JRR, Lingwood D, Kanekiyo M, Yassine HM, Ma SS et al (1950) 2015 H5N1 vaccine-elicited memory B cells are genetically constrained by the IGHV locus in the recognition of a neutralizing epitope in the hemagglutinin stem. J Immunol Baltim Md 195(2):602–10 10.4049/jimmunol.1402835 PMC4491024 26078272

[CR53] Zhang J, Kobert K, Flouri T, Stamatakis A (2014) PEAR: a fast and accurate Illumina Paired-End reAd mergeR. Bioinformatics 30(5):614–620 24142950 10.1093/bioinformatics/btt593 PMC3933873

[CR54] Zhou T, Lynch RM, Chen L, Acharya P, Wu X, Doria-Rose NA et al (2015) Structural repertoire of HIV-1-neutralizing antibodies targeting the CD4 supersite in 14 donors. Cell 161(6):1280–1292 26004070 10.1016/j.cell.2015.05.007 PMC4683157

[CR55] Zhu Y, Yang X, Ma C, Tang H, Wang Q, Guan J et al (2021) Antibody upstream sequence diversity and its biological implications revealed by repertoire sequencing. J Genet Genomics Yi Chuan Xue Bao 48(10):936–945 34420911 10.1016/j.jgg.2021.06.016

