# Supplementary material for: Novel polymorphic and copy number diversity in the antibody IGH locus of South African individuals
Source: Immunogenetics. 2024 Dec 4;77(1):6. doi: 10.1007/s00251-024-01363-7 (PMC11615098; doi:10.1007/s00251-024-01363-7)
Supplement: Supplementary file 1 — Supplementary file1 (DOCX 18 KB) [file 251_2024_1363_MOESM1_ESM.docx]

Novel Polymorphic and Copy Number Diversity in the Antibody IGH Locus of South African Individuals

Journal: Immunogenetics

Alaine A. Marsden^1,2^, Martin Corcoran^3^, Gunilla Karlsson Hedestam^3^, Nigel Garrett^4,5^, Salim S. Abdool Karim^4,6^, Penny L. Moore^1,2,4^, Dale Kitchin^1,2^, Lynn Morris^1,4^, and Cathrine Scheepers^1^

1. SA MRC Antibody Immunity Research Unit (AIRU), University of the Witwatersrand, Johannesburg, South Africa
2. HIV Virology Section, Centre for HIV and STIs, National Institute for Communicable Diseases (NICD), a Division of the National Health Laboratory Service (NHLS), Johannesburg, South Africa
3. Department of Microbiology, Tumor and Cell Biology, Karolinska Institutet, Stockholm, Sweden
4. Centre for the AIDS Programme of Research in South Africa (CAPRISA), University of KwaZulu Natal, Durban, South Africa.
5. Discipline of Public Health Medicine, School of Nursing and Public Health, University of KwaZulu-Natal, Durban, South Africa
6. Department of Epidemiology, Mailman School of Public Health, Columbia University, New York, USA

Corresponding Author:

Dr. Cathrine Scheepers - cathrine.scheepers@wits.ac.za

# **Supplementary Figures**

#### **Table 1: Primers for germline NGS.**

Primers designed to amplify germline IGHV genes, grouped by target subgroup. The number of genes expected to be amplified by a particular primer pair or grouping is indicated as well as the amplicon size expected to be produced.

#### **Table 2: IGHV Alleles Detected**

This table lists the genes and alleles detected in this study. For representation and analysis, alleles of genes with a designated "D" at the end were treated as alleles of the original gene without "D", e.g. IGHV2-70D and IGHV2-70. This is because these genes are duplications of the original gene. However, these alleles are preserved here so they can be matched with the correct database allele. The genes/alleles are also differentiated by source, either genomic or transcriptomic.

#### **Table 3: Identity of Novel IGHV Alleles**

A list of the novel alleles identified in this study, along with the dataset that the alleles were identified in, as well as the closest IMGT allele match for each sequence and the identity of the sequence to that allele match. The transcriptomic data has additional columns for the number of reads, the length distribution (minimum, maximum and mean) and the read error distribution (minimum, maximum and mean) of each inferred novel allele.

#### **Table 4: IGHV alleles with matching V regions showing mutations in other regions**

This table contains some examples of different IGHV alleles (column 1) which have identical V regions but differ in other parts of the gene. The variation is annotated with the region, if it matches another allele and how many SNVs are contained in that region which differ from the closest reference

#### **Table 5: IGHV Leader Sequences**

This table contains the leader sequences analysed in this study for novel variation.

#### **Table 6: Alleles of duplicated IGHV genes from genomic data**

The participants that contain duplications detected in the genomic sequencing of this study are listed here, with the alleles that contribute to the detection of a duplication. Of note, where there are alleles listed twice, this is because while the V-region designation for the alleles are identical the sequences differ in other regions of the gene.

#### **Table 7: RabHit output table**

The table details the haplotyping for each participant by gene. The table contains the alleles identified for each haplotype, denoted by IGHJ6*02 and IGHJ6*03. The proirs_row column denotes the priors based on relative allele usage of the anchor gene. The proirs_col column is denotes the priors based on relative allele usage of the inferred gene.The bayesian significance (k-value) for each allele is represented in each k column (k_value_allele_1...) with each number corresponding to the alleles in order from the alleles column. The counts (counts_allele_1...) column represent the number of reads that are associated to each haplotype for each allele, in order from the alleles column. The second sheet has a table noting the total read count, read length distribution (minimum, maximum and mean) and read error distribution (minimum, maximum and mean) for each allele in each participant that was haplotyped. The last comments column provides reasoning for the exclusion or inclusion of certain duplications.

#### 
